# A pragmatic effectiveness randomized controlled trial of the duration of psychiatric hospitalization in a trans-diagnostic sample of patients with acute mental illness admitted to a ward with either blue-depleted evening lighting or normal lighting conditions

**DOI:** 10.1186/s13063-019-3582-2

**Published:** 2019-08-01

**Authors:** Jan Scott, Knut Langsrud, Daniel Vethe, Kaia Kjørstad, Cecilie L. Vestergaard, Patrick Faaland, Stian Lydersen, Arne Vaaler, Gunnar Morken, Terje Torgersen, Håvard Kallestad

**Affiliations:** 1 Department of Mental Health, Norwegian University of Science and Technology, Trondheim, Norway; 2 Institute of Neuroscience, Newcastle University, Newcastle upon Tyne, UK; 3 Division of Mental Health Care, St. Olavs University Hospital, Trondheim, Norway; 4 Department of Research and Development, St. Olavs University Hospital, PO Box 3250, Sluppen, 7006 Trondheim, Norway

**Keywords:** Mental disorders, Acute treatment, Inpatient, Chronotherapy, Light, Blue-depleted light, Admission, Sleep, Circadian rhythms

## Abstract

**Background:**

There is increasing recognition of the need to stabilize sleep-wake cycles in individuals with major mental disorders. As such, clinicians and researchers advocate the use of interventions targeted at sleep and circadian dysrhythmias as an adjunct to the standard treatments offered for acute illness episodes of a broad range of diagnoses. To determine the trans-diagnostic generalizability of chronotherapy, we explore the benefits of admitting individuals with an acute illness episode to a psychiatric inpatient unit where changes in light exposure are integrated into the therapeutic environment.

**Methods/design:**

A two-arm, pragmatic effectiveness, randomized controlled treatment trial, where individuals admitted for acute inpatient psychiatric care will be allocated to a ward with blue-depleted evening light or to a ward with the same layout and facilities but lacking the new lighting technology. The trial will test whether the experimental lighting conditions offer any additional benefits beyond those associated with usual treatment in an acute psychiatric inpatient unit. The main objectives are to examine any differences between groups in the mean duration of hospitalization in days. Additional analyses will compare group differences in symptoms, functioning, medication usage, and side effects and whether length of stay is associated with stability of sleep-wake cycles and circadian rhythms. Ancillary investigations should determine any benefits according to diagnostic subgroups and potential drawbacks such as any adverse effects on the well-being of professionals working across both wards.

**Discussion:**

This unit offers a unique opportunity to explore how exposure to different lighting conditions may modify sleep-wake cycles and how any changes in sleep-wake cycle may impact on the clinical and functional outcomes of individuals experiencing an acute episode of a severe mental disorder that requires inpatient care. The findings could influence the future design of hospital units offering care to patients with mental or physical disorders.

**Trial registration:**

ClinicalTrials.gov, ID: NCT03788993. Retrospectively registered on 28 December 2018.

**Electronic supplementary material:**

The online version of this article (10.1186/s13063-019-3582-2) contains supplementary material, which is available to authorized users.

## Background

Recent decades have seen increased attention to the impact of disturbed sleep on general health [1]. For example, sleep-wake cycle abnormalities linked with circadian dysrhythmias are associated with physical disorders such as diabetes, obesity, and a greater risk of cancer, etc. [2]. As light is a central *zeitgeber* of the circadian system, some researchers have explored the benefits of phototherapy or chronotherapy for selected medical illnesses, especially in those individuals who have a concurrent comorbid mental disorder [3]. The latter is noteworthy as sleep problems are uniquely important in the field of mental health [4]. For instance, sleep abnormalities may be prodromal symptoms heralding the onset of a major mental disorder, sleep-wake cycle disruptions are criterion symptoms for diagnosing unipolar and bipolar disorders and circadian dysrhythmias may exacerbate suicidal behaviors [5]. Evidence from our research demonstrates that day-to-day variability in sleep-wake cycles is associated with longer duration of acute psychiatric admissions and frequency of aggression or violent incidents [6, 7]. Lastly, sleep problems are often the last symptoms to resolve during recovery from an acute episode of a mood or psychotic disorder [8, 9]. Overall, experimental and clinical research emphasize the reciprocal relationship between sleep-wake disruptions and mental disorders showing that they perpetuate and exacerbate each other and that improved sleep is associated with improvements in mental state [10–13].

The observations noted have increased awareness of the need to stabilize sleep-wake cycles in individuals with major mental disorders and highlighted the importance of providing interventions targeted at circadian dysrhythmias as an adjunct to other treatments for acute illness episodes [14]. Psychological and pharmacological interventions are efficacious approaches for sleep-wake cycle disturbances in adults without comorbid mental disorders [15–17]. However, their use in individuals with an acute exacerbation of a major mental illness can be problematic, including attenuation of the benefit-to-risk ratio for therapies or contra-indications to the use of some medications [15–17]. Partly as a response to these concerns, but also because of new research on circadian rhythms, attention has shifted to the potential role of interventions based on controlled exposures to environmental stimuli that act on biological rhythms [18]. These strategies initially focused on, e.g., morning bright-light therapy for seasonal affective disorders, sleep-wake disorders, and certain sub-types of depression; but there has been an increasing recognition of the importance of darkness at night to improve sleep-wake disorders and affective disorders.

Dark therapy is a treatment where the patient is in darkness an extended period (e.g., between 1800 h to 0800 h), and was initially described in two case-studies [19, 20] before it was tested in a clinical trial with bipolar patients in a manic episode [21]. Although the results were promising, clinical use has been limited as the patient have to be in complete darkness for about 12–14 h. Because the circadian effect of light on humans is primarily mediated by the intrinsically photoreceptive retinal ganglion cells (ipRGC) that have peak sensitivity to blue light [22], it was hypothesized [23], and later shown, that a clinical effect can also be achieved by specifically blocking the blue part of the light spectrum using blue-blocking glasses, rather than being in darkness [24, 25]. No severe side effects were reported, but two manic patients had emerging depressive symptoms that were diminished in less than 1 day [25]. Other trials have also used blue-blocking glasses at night as a treatment for sleep-wake disorders and affective disorders [26–29]. Still, these trials have been small and/or in homogeneous samples with a specified disorder, and have required the study participants to adhere to a protocol at specified times of the day (resting in forced darkness or wearing glasses).

The above represent interesting treatment advances. However, given the prevalence of sleep-wake cycle disturbances in individuals with mental disorders, it is logical to extend trials of the use of such interventions to broader trans-diagnostic populations. Further to enhance generalizability, it would help to avoid giving personal responsibility to individuals who are acutely unwell regarding the timing of their exposure to different intensities or spectra of light. A pragmatic alternative is to create a therapeutic environment where changes in light exposure are regulated automatically and where programmable lighting conditions form an integral part of a hospital unit. New light-emitting diode (LED) lights can be programmed to emit low levels of blue light which creates a blue-depleted light environment in the hospital in the evening and night. This is an intriguing option as, to date; little consideration has been given to how contemporary technology might be employed to augment any benefits of acute treatment in an inpatient facility. Historically, acute psychiatric admission units have offered asylum and a place of safety, whilst ward routines and structured activities help to reduce arousal, regularize sleep-wake cycle patterns and improve self-esteem, etc. However, the focus is primarily on physical and pharmacological treatments that reduce symptoms and suicidality, enhance social functioning and sufficiently improve the individual’s mental state to allow a timely return to outpatient or community care. Less attention has been given to the creation of a state-of-the-art inpatient milieu [30].

Our clinical and research staff has been involved in the planning and design of a newly built psychiatric unit and this process has allowed us to consider how the inpatient environment might be modified to try to enhance recovery from acute illness. The unit comprises of two wards: one ward incorporates state-of-the-art lighting technology whilst the other ward has an identical layout and facilities but has normal lighting conditions. This unit offers a unique opportunity to explore how exposure to different lighting conditions may modify sleep-wake cycles and how any changes in sleep-wake cycle may impact on the clinical and functional outcomes of individuals experiencing an acute episode of a severe mental disorder that requires inpatient care. The findings could influence the future design of hospital units offering care to patients with mental or physical disorders.

### Aims

We aim to recruit 400 individuals who give written informed consent to participate in a two-arm pragmatic effectiveness randomized controlled clinical treatment trial (RCT). Based on projected admission rates, we believe that this sample size is at the lower limit of the estimated study population that will be available for inclusion (as we are permitted to continue recruitment for at least six consecutive months).

Eligible individuals will be allocated to a ward with a lighting system that produces an environment with blue-depleted evening light or to a ward with the same layout and facilities but lacking the new lighting technology. The trial will test whether the environment with programmable lighting conditions offers any additional benefits beyond those associated with standard treatment in an acute psychiatric inpatient unit. The main objective is to examine if there is any difference between groups in the mean duration of hospitalization. Also, we will explore whether level of symptoms, functioning, episodes of suicidality or aggressive behavior, medication usage, and self-reported side effects differ between groups and whether a shorter duration of admission is associated with greater stability of sleep-wake cycles and circadian rhythms.

Given that this trial takes place in a unique setting, we will undertake several ancillary investigations to determine the range of benefits or adverse effects for subpopulations of patients (e.g., different diagnostic subgroups, etc.) and examine any potential benefits or drawbacks to the use of this new lighting system, including actigraphic recordings of sleep-wake patterns of nurses and any self-reported effects on well-being that are recorded by professionals who experience working in both wards.

## Methods/design

The protocol for the RCT follows the Standard Protocol Items for Randomized Trials (SPIRIT) Statement guidelines [31] and is registered at the ClinicalTrials.gov website with identifier: NCT03788993. The SPIRIT Checklist and other appropriate details are included as an online supplement (Additional file 1).

### Trial design, setting, and interventions

This is a single-center, unblinded, two-arm, parallel-group, pragmatic effectiveness RCT of differences in the mean duration of acute psychiatric hospitalization in days for individuals exposed to experimental lighting compared with normal lighting conditions.

The study is located at a newly built acute psychiatric unit at St. Olavs Hospital, Østmarka (Trondheim, Norway) which serves a catchment area of 300,000 people. The unit has 40 patient rooms divided equally between two wards that are built around two atria. Each hospital ward has the same layout and facilities with five rooms (25% of the total) designated as “psychiatric intensive care beds” (targeted at the most severely ill patients and offering a higher staff-to-patient ratio than the rest of the ward). The light intensity (photopic lux) is similar in both wards, but individuals are exposed to a different spectrum of light in each ward (e.g., melanopic lux is kept below 25 in the blue-depleted unit).

During the recruitment phase, patients admitted to the unit are randomized 1:1 to the experimental or control (normal) lighting conditions.

1. Experimental condition: a 20-bedded ward with tunable LED lamps. At 1800 h the lighting undergoes a 30-min transition during which the green and blue LEDs are dimmed to produce blue-depleted amber-colored lighting. At 0650 h a 10-min transition program changes the light color to normal indoor lighting (3000 K of color temperature) which then continues until 1800 h. The light intensity is dimmed to 20% (of the maximum) from 2300 h to 0650 h

As well as the LED system, blue-blocking window filters are deployed in the evening. All television sets have permanent blue-blocking filters and the outdoor area has external lights that block blue light. Use of electronic media is not restricted (unless access is limited in accordance with an agreed treatment plan), but patients are provided with blue-blocking screens that can be attached to the front of all electronic devices. If a patient leaves the blue-depleted unit after 1830 h they are offered blue-blocking glasses to wear. The amount of blue light in the ward was assessed prior to commencing the RCT. This demonstrated that the light exposure is well-matched to the amount shown in laboratory settings to minimally suppress melatonin [32, 33] and this was tested in an onsite pilot study with healthy adult volunteers (ISRCTN12419665).

2. Control condition: a 20-bedded ward with normal indoor lighting installed. The light intensity is dimmed to 20% (of the maximum) during the night (from 2300 h to 0650 h)

### Participants and procedure

**Fig. 1:**
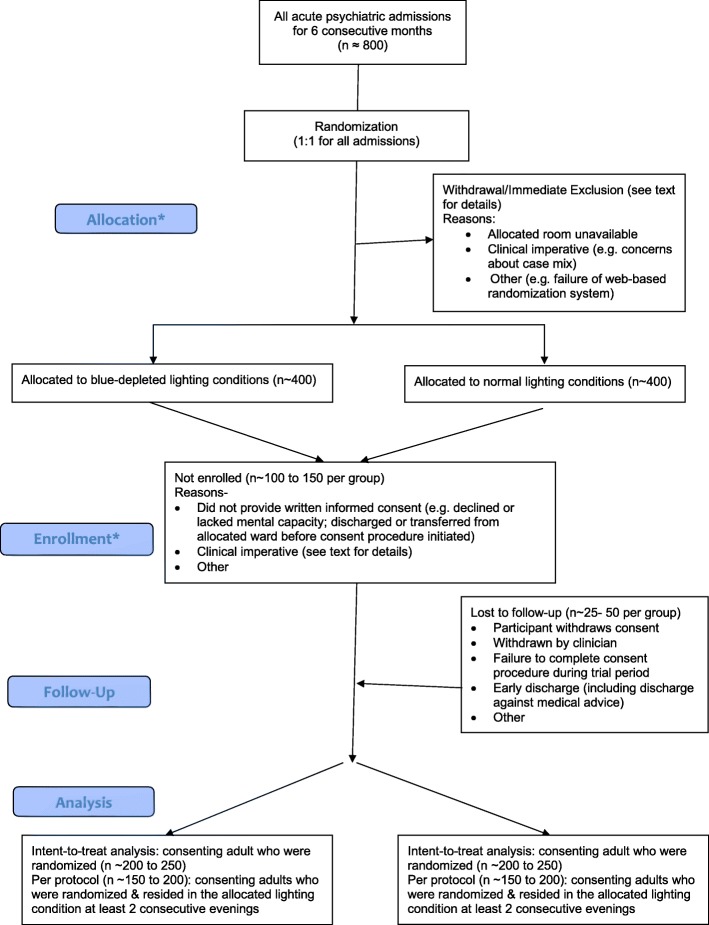
Study flowchart (*the study employs deferred consent, so allocation precedes enrollment)

The flowchart for the RCT is shown in Fig. 1.

#### Recruitment

Admissions occurring at any time during each 24-h period and on any day of the week throughout the 6-month recruitment period are considered eligible for study inclusion.

No specific recruitment strategy is employed, but should recruitment fail to reach the required sample size (for any reason) within an allocated 6-month time period, we will recommence recruitment in late 2019, i.e., all recruitment will be undertaken during months with similar daytime light exposure (contact HK for further details).

#### Eligibility criteria

A frequent barrier to research into acute psychiatric admissions is that, at the time of hospitalization, many individuals lack mental capacity to give informed consent (they may be severely ill, suicidal, lack insight or be hospitalized involuntarily) and/or clinicians and researchers may regard a discussion about research participation inappropriate or unethical. However, as noted in acute medicine research, excluding a large proportion of patients from a RCT purporting to investigate acute admissions can bias the included sample so that it is unrepresentative of the inpatient population and undermines the generalizability and real-world impact of any study findings [34]. For this reason, we did not employ any patient exclusion criteria but, with ethical approval, used a post-randomization, deferred (also known as delayed) consent procedure [34–36]. The RCT eligibility criteria are as follows:

##### Inclusion criteria

All individuals aged ≥ 18 years who are admitted to the acute inpatient unit during the study period are eligible for randomization. Any patients who are re-admitted during the study period are eligible for re-randomization.

##### Exclusion criteria

There are no pre-randomization exclusion criteria, but individuals may be withdrawn from the study immediately post randomization (see “Withdrawal criteria” below).

#### Randomization

Randomization is based on an allocation concealment procedure.

As soon as the decision to admit the patient is confirmed, the individual is randomly allocated 1:1 to one of the two arms of the RCT using a web-based randomization program with a variable block design. This occurs without any consultation with wards (regarding bed availability, case mix or staffing levels, etc).

The procedure was developed and is managed by the Unit for Clinical Research (at the Department of Medicine and Health, NTNU) and can be implemented at any time of the day or night. Randomization is undertaken by the nurse coordinating the intake process (who participated in pre-trial instruction and training), but they cannot influence the process in any way. When the intake nurse logs in to a web-based program, the randomization program issues an authentication code that is sent to the unit (a telephone back-up system is available should there be any problems with the web-based program).

#### Withdrawal criteria

As randomization occurs at the point of admission, all exclusions are de facto post randomization. Immediately post randomization, there are two potential reasons for withdrawal from the RCT:

1. Lack of availability of rooms (as allocated at randomization): acute wards operate at high levels of bed occupancy, so sometimes there will be no rooms available in the ward to which the individual is allocated (i.e., the randomization process cannot be completed)
2. Clinical imperative: sometimes a senior clinician may decide that it is inappropriate to admit an individual to the room to which they are randomized. The most frequent reasons for this decision are clinical concerns about (a) how this admission would affect the case mix within the ward (e.g., it may be inappropriate to co-locate several patients with acute mania) and/or (b) following through with the randomization process may compromise the safety, care or treatment of existing inpatients or of the individual being admitted (e.g., it may not be possible to provide the appropriate staff-to-patient ratio required if all individuals with higher levels of need are co-located)

During the admission withdrawal may occur because:

1. The individual is unwilling to give written informed consent at any time during their admission (when approached according to the deferred consent procedure) or is unable to give informed consent for the duration of the study (i.e., they remain persistently and severely ill and/or lack mental capacity)
2. The consent procedure was incomplete: an individual may be discharged early or have an unplanned discharge (so they may not be approached about study participation or have only given verbal, but not written consent)
3. A patient will be withdrawn from the study if they are absent for > 24 h from the ward to which they randomized (e.g., they may be transferred to a medical ward for several days; a patient may request or clinicians may instigate transfer to another ward; medical or nursing staff may decide that a patient should be transferred to the other ward participating in the RCT because of clinical, case mix or staffing issues, etc.)
4. An individual can decline to participate at any stage of the study and/or a mental health professional can recommend withdrawal of an inpatient from the RCT if they have any clinical concerns regarding an individuals’ participation (e.g., if they believe that a patient has experienced an adverse event associated with exposure to the blue-depleted light)

A record will be kept of all reasons for withdrawal.

### Assessments

Descriptions of all measures are provided below, and the timing of study assessments is summarized in Table 1.

**Table 1:** Targets and timing of assessments

Assessment	Collected via	Time			
		Intake/first 24 h	Daily	Discharge	Hospital records
Duration of admission in days	Data extracted from electronic records				X
Admission status (voluntary or involuntary)	Data extracted from electronic records	X			
Clinical diagnosis (including comorbid diagnoses)	Mental health professionals^a^	X		X	
Demography and clinical history (including current, past and forensic)	Mental health professionals^a^	X			X
Physical health (including anthropometrics)	Nurse	X			
Clinical Global Impression (CGI)	Mental health professionals^a^	X	X	X	
Clinical Global Impression-Severity (CGI- S)	Mental health professionals^a^	X		X	
Clinical Global Impression-Improvement (iCGI-I)	Mental health professionals^a^	X	X	X	
Risk of suicide	Mental health professionals^a^		X		
Risk of or actual aggressive behavior and associated interventions	Nurse		X		
Monitoring of sleep-wake cycle and daytime activity	Xethru radar sensor		X		
Prescribed medications	Data extracted from electronic records	X			X
Adherence to lighting condition	Nurse		X		
Satisfaction and perceived benefits	Study participant			X	
Side effects	Study participant			X	
Adverse events	Mental health professionals^a^		X		

See text for additional details of rating scales and assessments employed

^a^Primarily psychiatrists or clinical psychologists, but may include trained nurses and/or duty psychiatrist

A key consideration in the selection of assessment tools was that they were already used routinely or could easily be incorporated into ward procedures, and that clinical staff were familiar with, or had received training in, the use of the instruments. The design and setting of the study mean that it is unfeasible to blind patients, clinicians or investigators. However, we have used electronic hospital records and advanced technology to collect objective data on sleep-wake cycles.

#### Baseline demographic and clinical information

The intake assessment records detailed information on the following:

- Age, sex, ethnicity, marital status, living situation, years of education, employment status
- Current diagnosis or diagnoses (according to the *International Classification of Diseases 10*) [37]. As in our previous research, consensus expert opinion is employed to review all diagnoses at discharge. If the intake and discharge diagnoses differ, the latter is employed in analyses (as acute intake diagnoses may be less reliable)
- Type of admission (voluntary or involuntary), number of psychiatric admissions and total number of inpatient bed-days in the 2 years prior to the index admission and current prescribed medication
- Details of current presentation including evidence of sleep problems (during the preceding month), level of functioning, alcohol and substance misuse, risk of or actual harm to self or others, current physical health
- Past psychiatric and forensic history, and history of comorbid medical illnesses

#### Primary outcome

The primary outcome measure is mean duration of admission in days per individual. Admission is defined as the date and time of commencement of the intake assessment (recorded electronically when the randomization code is generated); discharge is defined as the date and time that the patient left the lighting condition to which they were randomized for > 24 h.

#### Secondary outcomes

Secondary outcomes are focused on clinical changes over time and treatments or other interventions reported during admission.

- Clinical assessments

i. Objective assessments:

Sleep-wake cycle: individual sleep and activity patterns will be assessed using de-identified data collected via radar (Xethru sensors) installed in each room. The sensor is a low-powered ultra-wideband radar that allows contact-free assessment of sleep-wake patterns with high accuracy, sensitivity, specificity, and Cohen’s kappa compared with polysomnographic (PSG) recordings (mean values 0.93, 0.96, 0.70, and 0.67, respectively) [38].

Employing best available scoring algorithms, raw data from daily recordings will be used to estimate total sleep time (TST), sleep onset latency (SOL), number of nocturnal awakenings, wake after sleep onset (WASO), and final wake time for each participant, along with day-to-day variability in sleep onset, sleep offset, and TST.

ii. Observer assessments

The Clinical Global Impression Scale (CGI) is rated on a 1–7 scale (high scores indicate worse clinical or functional status) and is a well-established outcome measure in trans-diagnostic studies that has moderate to high correlation with disorder specific assessments, both self-reported and clinician administered (e.g., Hamilton Rating Scale for Depression, the Montgomery-Asberg Depression Rating Scale, the Beck Depression Inventory, Hamilton Rating Scale for Anxiety, Positive and Negative Syndrome Scale, Leibowitz Social Anxiety Scale, Brief Psychiatric Rating Scale, Scale for the Assessment of Negative Symptoms) [39, 40].

In this RCT, psychiatrists, psychologists, and nursing staff trained in the use of the CGI, will record a consensus score for each study participant during the daily ward meetings. During the weekends, the psychiatrist on duty and nursing staff will provide the CGI consensus ratings.

The CGI ratings will be based on all available information (nurse observations, clinical assessments, hospital medical records, etc.) and will be used in two ways in this RCT: (a) to monitor day-to-day changes in mental state and functioning, and (b) to record overall change from admission to discharge.

- Clinical Global Impression, Severity subscale (CGI-S) is a Likert scale ranging from 1 to 7 (from “Normal, not at all ill” to “Among the most extremely ill patients”) [39]. The CGI-S ratings are benchmarked relative to the total inpatient population and are scored on two occasions only: the morning after admission to the unit and at discharge (based on the preceding 24 h). The intraclass correlation coefficient of CGI-S between independent raters have been shown to be 0.64 for the initial assessment and 0.70 for a second assessment after 14 days [41]
- Clinical Global Impression, Improvement subscale (improved version: iCGI-I) captures change over time with ratings ranging from − 6 (maximum deterioration) to + 6 (ideal improvement) [41]. The iCGI-I is used (a) to monitor day-to-day changes in mental state and functioning, and (b) to record overall change from admission to discharge. The intraclass correlation coefficient of iCGI-I between independent raters has been shown to be 0.74 [41]
- Risk of Harm to Self or Others: suicide risk is assessed daily (rated according to level of risk and/or need of continuous observation) and risk of aggressive behavior is assessed three times per 24 h using the Brøset Violence Checklist (BVC) [42, 43]. The BVC is a six-item scale and the sum score indicates risk of violence (low = 0) and has acceptable interrater reliability (kappa = 0.41 for the sum score) and specificity (Area Under the Curve of Receiver Operating Characteristics = 0.82) [44]. Incidents of aggressive behavior will be systematically recorded using the Staff Observation Aggression Scale-Revised (SOAS-R) [45] and interventions employed will be recorded. The SOAS-R has acceptable interrater reliability (between kappa = 0.61 to kappa = 0.74) [46]

b) Treatments and interventions

i. Medications: daily doses and classes of medications or other treatments or interventions prescribed per individual during admission will be recorded
ii. Change in admission status: if a patient is admitted involuntarily, we will record the time until their status is reclassified as voluntary (as a marker of improved insight and mental capacity). Similarly, for some individuals time to change from voluntary to involuntary status will need to be recorded

#### Patient-related experiences and other outcomes

For everyone involved with the new unit, a key aspiration is to try to capture information regarding patient experiences of the experimental condition and to evaluate benefits and harms. In this RCT, we will assess acceptability (adherence, perceived satisfaction, and benefits) or harms (side effects and adverse effects) using observer and self-rated assessments.

- Adherence

For individuals allocated to the blue-depleted conditions, adherence with the intervention is assessed using an item checklist to record any exposure to normal lighting (duration and reasons), whether blue-blocking glasses were worn as appropriate (e.g., when exiting the unit) and whether blue-blocking filters were employed on media devices

b) Satisfaction and benefits

Mean levels of patient satisfaction with an admission are routinely assessed using the standard patient satisfaction questionnaire completed at discharge. The questionnaire was developed by the Norwegian Institute of Public Health, is used throughout the Norwegian Health Care system and consists of 10 items scored on a 5-point Likert scale (1 = low satisfaction). Some items are relevant to examining experiences of the different lighting conditions, side effects (see below) and perceived benefit of the admission (1 = no benefit)

c) Side effects and adverse events

The frequency of any side effects or adverse events experienced by individuals admitted to each lighting condition will be recorded using the eight-item Headache and Eye Strain Scale (HES), which has been shown to be sensitive to exposure to different lighting conditions [47]. This is supplemented by eight items that may reflect side effects of acute psychiatric treatments (e.g., dizziness, gastro-intestinal disturbances, daytime sleepiness, poor sleep quality, and restlessness, etc.). Each symptom is rated on a 4-point scale (ranging from absent to severe).

To capture any putative adverse events experienced during the admission we will record the occurrence of any serious or untoward incidents in each ward (such as non-accidental and accidental deaths, near fatal events, severe violence, etc.). Also, we will note if any patients are transferred out of the blue-depleted light environment because of clinical opinion suggesting that it is having a detrimental effect on the individual.

#### Other outcomes

Several ancillary studies are planned. For example, ethical approval has been granted to undertake an additional study (running concurrently with the RCT) to examine the experiences of clinical staff who rotate their work schedules between the two wards (ISRCTN21603406), including sub-studies of subjective effects on sleep, cognition, and well-being, and objective actigraphic recordings undertaken during exposure to each lighting condition. Data regarding any side effects may be compared across staff and patients, but other studies will be reported separately.

### Sample size

The power calculation and sample size were estimated for the primary outcome measure, namely mean number of days hospitalized per individual exposed to the experimental or control lighting conditions. This outcome was chosen as hospitalization represents a major life event for patients, is the largest contributor to cost of care across psychiatric diagnoses, and it offers a proxy measure of any adjunctive benefits associated with the experimental lighting conditions over and above any gains associated with usual inpatient treatment.

The hospital database showed that there were 1639 acute psychiatric admissions between May 2016 and April 2017, with a mean length of stay of 6.3 days (range 1–158 days; total occupied patient bed-days per annum > 10,000). Assuming that the experimental lighting conditions lead to a reduction in the mean length of stay from about 6 to 5 days (with a standard deviation of about 3.5 days), then 194 participants in each condition will give an 80% chance at an alpha = 0.05 to detect a difference in the length of stay of 1 day using an intent-to-treat (ITT) analysis (and > 85% power to detect a reduction of 1000 patient bed-days occupied over the course of the year). We have assumed that there will be about 800 admissions to the unit over 6 months, and that post-randomization exclusions (lack of consent, etc.) and sample attrition (e.g., due to early discharge) will amount to about 30% (*n* = 240). Whilst the sample size required is 400 individuals (who give written informed consent), recruitment will continue for the entire 6-month period, and we will continue to randomize all admissions. As such, it is possible that we will be able to include up to 500 individuals.

### Data analysis plan

#### Data management

Data retrieved from electronic hospital records and from assessments will be de-identified and the files will be stored on secure data storage servers.

#### Statistical analyses

The primary statistical analyses will be performed by an independent statistician who is masked to the lighting conditions experienced by each group.

We will use a linear mixed-model analysis to examine the difference in mean duration of admission in days per individual according to group. As this primary outcome is heavily skewed and not normally distributed, we will use bootstrapping. The main analyses will be based on the ITT population (i.e., all individuals who gave consent and were randomized). Per-protocol (PP) analyses will be limited to participants who are hospitalized for at least two consecutive evenings in the inpatient unit. There will also be some individuals who were not randomized but who give informed consent for the use of some data in additional analyses.

We use a block randomization with random block length (which avoids adding to the burden of the intake process or delaying the admission procedure). As such, we will adjust for a pre-specified subset of demographic and clinical covariates (selected from those listed in the baseline assessments) which are known predictors of duration of a psychiatric admission: these are age, sex, diagnosis, presence of comorbidities, status at admission (involuntary or voluntary), and number of bed-days in the previous 2 years [48, 49]. It should be noted that some patients will be re-admitted and re-randomized during the study period, so we will use a two-level model (with admissions within patient as level 1 and patient as level 2).

For the analysis of the primary outcome, we expect complete data on duration of admission. For analyses of secondary outcomes, we will use multiple imputations to handle missing data as appropriate. We plan to use a mixed model with random intercept and random slope for secondary analyses which have one or more values per admission. Additional analyses will include subgroup (e.g., length of stay according to diagnostic groups; use of medications according to group, etc.) and exploratory analyses (e.g., examining whether improvement in sleep variables mediates duration of admission and risk of harm to self or others; prevalence of self-reported side effects in patients compared to nurses).

### Study monitoring

A Data and Safety Monitoring Committee will meet weekly to oversee the study progression, technical issues, and the safety of patients. The committee is comprised of the investigators involved with recruitment, clinical representatives from the unit (mainly psychiatrists and nurses), and statistical advisors; other representatives will be co-opted as required.

The trial sponsor routinely audits of one or more of the ongoing projects each year (selection may be at random or can be via a specified procedure). This monitoring ensures that the trial adheres to the protocol, procedures, and ethical standards and provides independent oversight and quality assurance. No interim analyses are planned.

### Patient and public involvement

Patient and public involvement has been a feature of the development of the inpatient service and the proposed research. For example, the leader of the User Group for Mental Health, St. Olavs Hospital, has reviewed the project and offered public support. Also, representatives of the advocacy group have been involved in all the processes related to research at the new unit from the start and have had several meetings with the research group that discussed the study design. Ongoing advice and support have been provided by medical and nursing colleagues working at the unit and others working at St. Olavs Hospital. This included dialog about what assessments could be incorporated into ward routines and the training needs of staff involved in the projects. International experts were consulted regarding the procedure for deferred consent and others offered advice regarding the program for and delivery of the chronotherapeutic interventions.

### Dissemination

There is considerable international interest in the lighting technology and the use of this program as an adjunct to standard treatment in an inpatient environment. As well as publication of findings regarding primary and secondary outcomes, we will publish descriptive articles, present information about the unit and the RCT findings at national and international conferences and will allow site visits by clinicians, researchers and patient advocates who wish to view the unit. The investigators will adhere to international guidelines regarding multi-authorship of manuscripts. No data will be released until all key outputs have been accepted and published in peer-review journals. However, interested parties can contact the senior investigator to discuss data-related issues.

## Discussion

There are several significant challenges to undertaking a pragmatic effectiveness RCT in an emergency or acute care setting. The broad range of reasons for acute admission and heterogeneity of psychiatric diagnoses included in the RCT necessitated the selection of a clinically meaningful primary outcome measure and of assessment procedures that minimized any research-related burden placed on patients or clinicians. Whilst an ITT approach is essential to any RCT, there is likely to be considerable “noise” in this primary analysis (e.g., patients may discharge themselves against medical advice; others are admitted in a crisis and then discharged within 24 h). As such, we anticipate that the PP analyses will be important in enhancing understanding of specific patient subgroups that may benefit from exposure to the novel lighting condition, and whether anyone experiences adverse effects. Additional planned analyses should offer important insights into putative benefits or issues that may influence whether other units introduce similar lighting systems in the future. Lastly, we are mindful that the lighting parameters are based on data obtained from healthy controls, and that we may need to further modify the program for exposure to blue-depleted light to optimize benefits for individuals with acute mental disorders.

## Trial status

The trial is ongoing and started patient recruitment on 23 October 2018. The study is expected to continue until the end of December 2019. No important protocol amendments are anticipated. However, if any would become necessary, the senior investigator or deputy would be responsible for communicating this to other investigators, the Ethics Committee, grant bodies, etc. Any important changes would be included in the study listing on the website for clinical trials in the Norway so that future participants would be aware of any new eligibility criteria. Protocol versions: the first version of the protocol was approved by the Ethics Committee on 6 June 2018; with two minor amendments submitted on 17 October 2018 and 19 December 2018. This is the second and final version approved on 4 January 2019.

## Additional file

Additional file 1: Standard Protocol Items for Randomized Trials (SPIRIT) 2013 Checklist for the protocol. (DOCX 35 kb)

## Data Availability

Not applicable. The manuscript does not contain any data.

## Abbreviations

BVC: Brøset Violence Checklist
CGI: Clinical Global Impression Scale
CGI-S: Clinical Global Impression Scale, Severity subscale
HES: Headache and Eye Strain Scale
iCGI-I: Improved Clinical Global Impression Scale, Improvement subscale
ITT: Intention to treat
LED: Light-emitting diode
NTNU: Norwegian University of Science and Technology
PP: Per protocol
PSG: polysomnography
RCT: Randomized controlled clinical treatment trial
REK: Regional Etisk Komité (Regional Ethical Committee).
SOAS-R: The Staff Observation Aggression Scale-Revised
SOL: Sleep onset latency
SPIRIT: Standard Protocol Items for Randomized Trials
TST: Total sleep time
WASO: Wake after sleep onset
